# Transcutaneous Vagus Nerve Stimulation Combined with Robotic Rehabilitation Improves Upper Limb Function after Stroke

**DOI:** 10.1155/2017/7876507

**Published:** 2017-12-10

**Authors:** Fioravante Capone, Sandra Miccinilli, Giovanni Pellegrino, Loredana Zollo, Davide Simonetti, Federica Bressi, Lucia Florio, Federico Ranieri, Emma Falato, Alessandro Di Santo, Alessio Pepe, Eugenio Guglielmelli, Silvia Sterzi, Vincenzo Di Lazzaro

**Affiliations:** ^1^Unit of Neurology, Neurophysiology, Neurobiology, Department of Medicine, Università Campus Bio-Medico di Roma, Via Álvaro del Portillo 21, 00128 Rome, Italy; ^2^Fondazione Alberto Sordi-Research Institute for Ageing, Via Álvaro del Portillo 5, 00128 Rome, Italy; ^3^Unit of Physical and Rehabilitation Medicine, Department of Medicine, Università Campus Bio-Medico di Roma, Via Álvaro del Portillo 21, 00128 Rome, Italy; ^4^San Camillo Hospital IRCCS, Venice, Italy; ^5^Unit of Biomedical Robotics and Biomicrosystems, Department of Engineering, Università Campus Bio-Medico di Roma, Via Álvaro del Portillo 21, 00128 Rome, Italy

## Abstract

The efficacy of standard rehabilitative therapy for improving upper limb functions after stroke is limited; thus, alternative strategies are needed. Vagus nerve stimulation (VNS) paired with rehabilitation is a promising approach, but the invasiveness of this technique limits its clinical application. Recently, a noninvasive method to stimulate vagus nerve has been developed. The aim of the present study was to explore whether noninvasive VNS combined with robotic rehabilitation can enhance upper limb functionality in chronic stroke. Safety and efficacy of this combination have been assessed within a proof-of-principle, double-blind, semirandomized, sham-controlled trial. Fourteen patients with either ischemic or haemorrhagic chronic stroke were randomized to robot-assisted therapy associated with real or sham VNS, delivered for 10 working days. Efficacy was evaluated by change in upper extremity Fugl–Meyer score. After intervention, there were no adverse events and Fugl–Meyer scores were significantly better in the real group compared to the sham group. Our pilot study confirms that VNS is feasible in stroke patients and can produce a slight clinical improvement in association to robotic rehabilitation. Compared to traditional stimulation, noninvasive VNS seems to be safer and more tolerable. Further studies are needed to confirm the efficacy of this innovative approach.

## 1. Introduction

Upper limb impairment is a common consequence of stroke with a deep impact on patient's quality of life. Since the efficacy of standard rehabilitative therapy is limited, alternative strategies are needed. Robot-assisted rehabilitation can be useful in stroke patients because it allows an intensive as well as task-specific training characterized by high repetition of movements in a strongly motivating environment [1–3]. Several studies have explored the possibility to potentiate the effect of robotic therapy by the association with noninvasive human brain stimulation techniques, such as repetitive transcranial magnetic stimulation (rTMS), that can induce neuroplasticity via long-term potentiation-/depression- (LTP-/LTD-) like phenomena [4]. Although intriguing, the evidence in support of this strategy remains low [5, 6]. Indeed, the literature analysis of the published data seems to demonstrate that the association of rTMS with robotic training has the same clinical gain derived from robotic therapy alone. Moreover, rTMS is contraindicated in patients who suffered from haemorrhagic stroke for the risk of inducing seizures [7]. For these reasons, there is great interest in the development of alternative techniques of neuromodulation that can foster the effect of robotic therapy.

Vagus nerve stimulation (VNS) is approved as adjunctive treatment for refractory epilepsy and depression but is currently under investigation for a wide range of neurological diseases [8]. In particular, recent studies have demonstrated that VNS paired with rehabilitation significantly improves forelimb strength and movement speed in rat models of ischemic [9] and haemorrhagic stroke [10]. VNS is believed to enhance the benefits of rehabilitation by promoting neuroplasticity [11]. Preliminary data [12] have showed that such approach is also feasible in patients; however, the diffusion of this technique is limited by its invasiveness. Indeed, VNS requires the surgical implantation of a stimulator of the cervical branch of the vagus nerve. Recently, it has been proposed a noninvasive technique that consists of transcutaneous stimulation of the vagus nerve (tVNS) in external auditory channel at the inner side of the tragus. Both neuroimaging [13] and neurophysiological [14] studies have demonstrated that the effect of tVNS on brain activity is quite similar to the effect induced by traditional, invasive VNS.

The aim of the present study was to explore whether tVNS can enhance the benefit induced by robotic rehabilitation on motor function of the upper limb in chronic stroke. Safety and efficacy of this combination have been assessed within a proof-of-principle, double-blind, semirandomized, sham-controlled trial.

## 2. Material and Methods

The study was performed accordingly to the Declaration of Helsinki and was approved by the Local Ethics Committee. The study was proposed to patients attending the outpatient clinic for cerebrovascular disorders of Campus Bio-Medico University Hospital. Inclusion criteria were as follows: (a) first-ever, ischemic or haemorrhagic stroke at least 1 year earlier; (b) hand function impairment; (c) and ability to give informed consent and comprehend instructions. Exclusion criteria were as follows: (a) previous surgical intervention on vagus nerve; (b) low hearth rate (<60 bpm); (c) cognitive impairment or any substantial decrease in alertness, language reception, or attention that might interfere with understanding instructions for motor testing; (d) apraxia; (e) excessive pain in any joint of the paretic extremity; (f) advanced liver, kidney, cardiac, or pulmonary disease; (g) history of significant alcohol or drug abuse; (h) depression or use of neuropsychotropic drugs such as antidepressants or benzodiazepines; (i) and pregnancy.

Fourteen patients with either ischemic or haemorrhagic chronic stroke were randomized to robot-assisted therapy associated with real or sham tVNS, delivered for 10 working days. Efficacy was evaluated by change in upper extremity Fugl–Meyer assessment (FMA) score. To assess safety, during the stimulation, heart rate (HR) and blood pressure (BP) were monitored. Moreover, to test the tolerability of tVNS, subjects were questioned about the presence of unpleasant sensations or other discomforts. Each day, patients received a session of robotic therapy immediately following the real or sham stimulation. All patients were evaluated at baseline (baseline) and just after the two weeks of treatment (post). At baseline, to evaluate neurological impairment and disability, we also included the following scales: National Institute of Health Stroke Scale (NIHSS), Rankin Scale, Barthel Index, and Modified Ashworth Scale. Spasticity was assessed by Modified Ashworth Scale at four different joints of affected arm: the shoulder, elbow, wrist, and fingers. For each patient, a cumulative score was obtained by summing the scores obtained in the four joints. The cumulative score ranges from 0 (no spasticity) to 16 (maximum spasticity, i.e., score 4 in all the considered joints).

The stimulation of the auricular branch of the vagus nerve was performed through an electric stimulator (Twister—EBM) and two Ag-AgCl electrodes (5 mm in diameter) placed in the left external acoustic meatus at the inner side of the tragus. For sham stimulation, electrodes were attached to the left ear lobe, an anatomical area that is outside the innervation of the auricular branch of the vagus nerve. tVNS was delivered as trains lasting 30 s and composed by 600 pulses (intratrain pulse frequency = 20 Hz; pulse duration = 0.3 ms) repeated every 5 min for 60 min. The intensity of stimulation was individually adjusted to a level ranging above the detection threshold and below the pain threshold. To reduce the risk of cardiac side effects, only the left ear was stimulated because vagal fibers to the heart are supposed to originate from the right side [15].

Robotic therapy was delivered at proximal or at distal segment of the affected limb according to the degree of impairment and the choice of the physician. The InMotion2 shoulder-elbow system (Interactive Motion Technologies Inc.) [16] was used for proximal limb segments and the InMotion3 wrist system (Interactive Motion Technologies Inc.) [17] for the treatment of distal segments. InMotion2 robot consists of a direct-drive mechanism that provides two translational degrees of freedom for elbow and forearm motion. Robot movement is enabled thanks to an impedance control that guides or perturbs the patient's movement. The InMotion3 robot enables unilateral wrist training characterized by low endpoint inertia and friction. Flexion-extension and radial-ulnar deviation are guaranteed thanks to two side-mounted actuators connected to a differential mechanism, while pronation-supination is actuated by another DC motor. An impedance control is implemented to assist patient's movement. Each day of robotic treatment consisted of three sessions of 320 assisted point-to-point movements, from the center to eight outbound targets, interspersed by four sessions of 16 unassisted recorded point-to-point movements. Robot assistance at each session was tuned on patients' performance during the 16 point-to-point sessions. During training, patients were required to move with a self-paced speed in a maximum time slot of 3 s. Robotic treatment was delivered daily for 10 consecutive working days, immediately after the end of real or sham tVNS. A physical and rehabilitation medicine doctor attended and assisted patients during treatment. During the intervention period, patients did not receive any additional physical therapy. Pharmacological therapy was also unchanged. Researchers randomizing patients and researchers delivering tVNS were not involved in outcome assessments and data analysis; moreover, rehabilitation doctors, patients, and researchers involved in data analysis were blind to the type of tVNS delivered (i.e., sham or real), in order to obtain a double-blind study design.

### 2.1. Statistics

Statistical analysis was performed using the IBM SPSS Statistics (Ver. 24). After checking that the baseline clinical measures were not different between groups, postintervention FMA of the two groups was expressed as percentage of baseline scores and compared by means of Mann–Whitney test.

In order to assess the safety of the stimulation, we measured systolic blood pressure, diastolic blood pressure, and heart rate, before and after each stimulation, every day, for ten days. For each of these measures, we performed a mixed-model repeated ANOVA with days (ten levels) and prepost (2 levels) as within subject factors and group (two levels: real and sham) as between subject factor. Correction for sphericity violations and multiple comparisons were applied as needed.

## 3. Results

Seven patients were randomized to robot-assisted therapy associated with real tVNS and seven patients to robot-assisted therapy associated with sham tVNS. One sham patient withdrew consent before the first session of treatment. Another sham patient withdrew because of difficulty in reaching the hospital after the second day of treatment. Data of these patients were not included in the analysis. Thus, a total of 12 patients completed the study: 7 real (mean age: 53.7 ± 15.6 years, 4 males) and 5 sham (mean age: 55.6 ± 15.9 years, 3 males). The real and sham groups were not significantly different regarding age, sex, type of stroke (haemorrhagic versus ischemic), and side of lesion. Time elapsed from stroke onset and clinical status at baseline (in particular, FMA score) were different between the two groups, but this difference was not statistically significant (*p* > 0.200 consistently) (Table 1).

**Figure 1:**
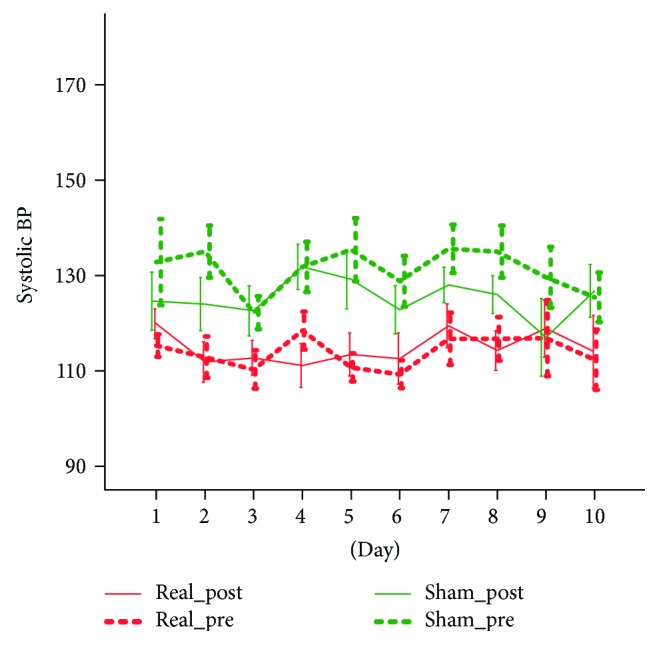
The effect of tVNS on systolic BP.

**Figure 2:**
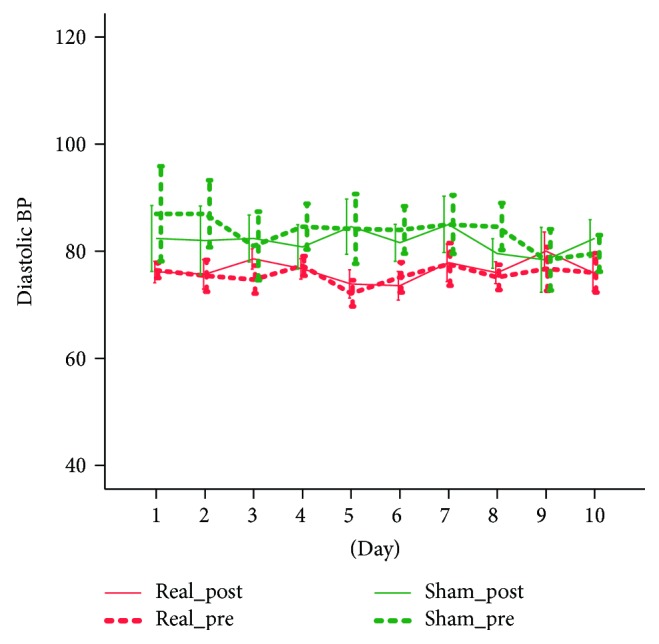
The effect of tVNS on diastolic BP.

**Figure 3:**
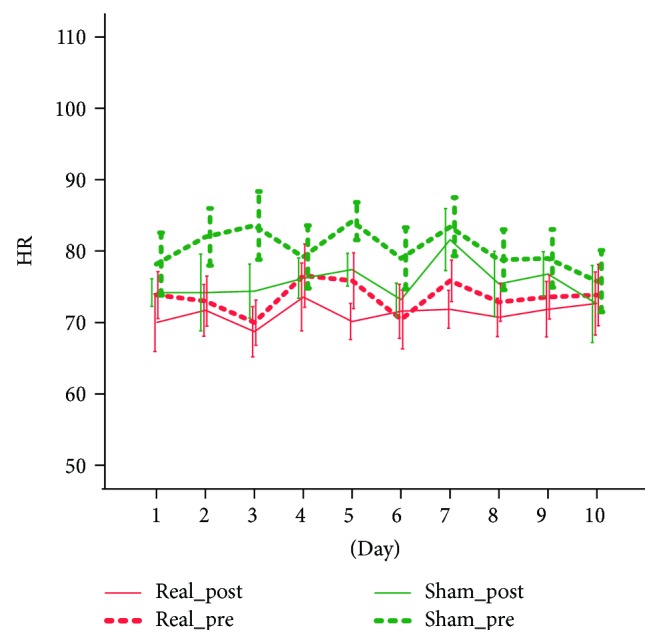
The effect of tVNS on HR.

The treatment was safe and tolerable. There were no adverse events, unpleasant sensations, or other discomforts. None of the patients required to stop stimulation. For systolic BP, the ANOVA mixed model showed no significant main effects nor significant interactions with the factor days (*p* > 0.200 consistently). We however found a significant prepost by group interaction (*F*(1.9) = 7.335, *p* = 0.024) which was largely related to the intergroup systolic pressure difference (*F*(1.9) = 9.986, *p* = 0.012), as no prepost significant differences were found within each group. This analysis unveiled that the two groups had an average significant systolic blood pressure differences but the stimulation has no significant effect on this parameter (Figure 1). Similar behaviour showed the diastolic blood pressure, for which we only found a significant prepost ^∗^group interaction (*F*(1.9) = 7.328, *p* = 0.024). No significant group differences nor prepost differences in each group were found (Figure 2). For the HR measure, we only found a significant prepost main effect (*F*(1.9) = 32.497, *p* < 0.001) that was confirmed in both groups and corresponded to a mild and not clinically relevant reduction of heart rate (2.3 bpm in the real group and 4.7 bpm in the sham group) (Figure 3).

**Figure 4:**
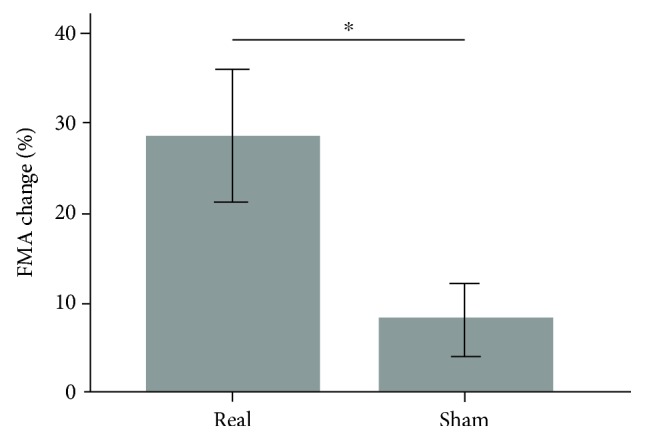
Effect of tVNS on FMA scores. The FMA score improved significantly (^∗^*p* = 0.048) more in the real group than in the sham group. FMA is expressed as percentage change with respect to baseline.

After intervention, FMA scores were significantly better in the real group as compared to the sham group (Mann–Whitney *U* = 5.00, *p* = 0.048) (Figure 4). Individual data, including the kind of treatment (robotics and VNS), the intensity of VNS, and the changes in FMA, HR, and blood pressure are reported in Table 2.

## 4. Discussion

This is the first study that has evaluated the feasibility of tVNS in chronic both ischemic and haemorrhagic stroke patients. Our data demonstrate that tVNS is safe and, combined to robot-assisted rehabilitation, can induce a slight but significant improvement of arm functionality. The treatment was well tolerated, and no adverse events or discomforts were reported from patients. In particular, we have not recorded any side effect that can occur with invasive VNS such as vocal cord palsy, dysphagia, nausea, taste disturbance, hoarseness, or neck tingling [12]. Since vagus nerve influences cardiac activity [18], we have carefully monitored HR and BP during tVNS session in order to identify any potential cardiovascular harm. We have not observed any clinically significant change in cardiovascular parameters throughout the stimulation. A slight and asymptomatic reduction of HR was observed both in the real and in the sham groups. Because this was present in both groups, it was not related to tVNS and thus represents an unspecific change that might be related to different causes such as patient relaxation during the course of the study. In a previous, randomized, placebo-controlled, double-blind study on ten healthy subjects [14], we have showed that tVNS does not change HR and BP. Similar results have been obtained from Shim et al. [19] that have treated thirty patients with refractory chronic tinnitus. Taken together, these findings suggest that tVNS is a safe technique that does not negatively influence cardiac functionality and can be used in stroke patients.

Even though this was a proof-of-principle study mainly aimed to demonstrate the feasibility of tVNS, our results suggest that the combination of vagal stimulation and robotic rehabilitation can improve arm functionality in chronic stroke patients. Indeed, both real and sham patients improved after the intervention but the change in FMA was significantly higher for the real group (5.4 versus 2.8 points; *p* = 0.048). This change, although slight, is considered clinically significant in chronic patients [20], especially in those with severe impairment of upper limb function [2]. A potential benefit of invasive VNS in chronic stroke has been recently described both in animal model [21] and in patients [12]. In a rat model of chronic stroke, Khodaparast et al. [21] have demonstrated that VNS paired with rehabilitative training significantly improves recovery of forelimb function compared to rehabilitation alone. Interestingly, ischemic lesion size is not reduced by VNS. According to the authors, this finding suggests that, in chronic stroke, VNS promotes recovery through a mechanism independent of neuroprotection, most likely by inducing neuroplasticity. This idea is further supported by additional experimental data showing that VNS increases levels of brain-derived neurotrophic factor (BDNF) and neurotransmitters such as noradrenaline linked to neuroplasticity and recovery after brain lesion [22, 23].

The feasibility of VNS in chronic stroke patients has been recently evaluated by a clinical trial involving twenty-one patients randomized to VNS plus rehabilitation or rehabilitation alone [12]. VNS has been performed by a surgically implanted device producing stimulation paired with rehabilitative exercises. The authors reported some minor adverse effects related to stimulating device, but no serious adverse events were observed. Arm functionality, measured by FMA, improved in both groups but more in the VNS group (between-group difference, 5.7 points). In this trial, VNS was delivered simultaneously with the rehabilitative training. Indeed, animal studies [21] have demonstrated that the timing of VNS-rehabilitation coupling is essential because recovery does not improve when VNS follows rehabilitation. This result supports the idea that the synergistic effect of VNS and rehabilitation depends on neuroplasticity, a timing-dependent phenomenon. Our study extends this concept demonstrating that also noninvasive VNS delivered before rehabilitation can ameliorate arm functionality. As described for rTMS, tVNS could increase the effect of rehabilitation by producing a priming effect on subsequent motor training [24].

Although intriguing, the results of our study, in particular the effect on FMA, should be considered cautiously. Indeed, the present study has some important limitations such as the small sample size, the use of different kind of robotic training, and the lack of a long-term follow-up.

## 5. Conclusions

Our pilot study confirms that VNS is feasible and safe in stroke patients and can produce a slight clinical improvement in association to robotic rehabilitation. Compared to traditional, invasive stimulation, tVNS seems to be safer and more tolerable. Further studies are needed to confirm efficacy and unveil the mechanisms of action of this innovative approach.

## Figures and Tables

**Table 1:** Demographic and clinical characteristics of the patients at baseline.

	Real (*N* = 7)	Sham (*N* = 5)	*p* value
Age (years)	53.71 ± 5.88	55.60 ± 7.12	1.00^a^
Sex (M)	4	3	0.447^b^
Months since stroke	93.71 ± 38.81	46.00 ± 21.85	0.432^a^
Fugl–Meyer	22.29 ± 3.51	32.60 ± 6.43	0.268^a^
NIHSS	6.14 ± 1.50	4.80 ± 0.74	0.639^a^
Barthel Index	72.14 ± 9.81	81.00 ± 9.00	0.639^a^
Modified Rankin	2.86 ± 0.40	2.20 ± 0.58	0.432^a^
Modified Ashworth Scale cumulative score	6.86 ± 1.16	5.40 ± 1.32	0.343^a^

All data are expressed as mean ± standard error. ^a^Mann–Whitney test; ^b^chi-square test.

**Table 2:** Effect of treatment on upper limb functionality and cardiovascular parameters.

Patient	Age	Gender	Stroke	Type of robot	Stimulation	VNS range intensity	FMA PRE	FMA POST	HR PRE	HR POST	sBP PRE	sBP POST	dBP PRE	dBP POST
1	44	M	Isch	InMotion2	Real	2.5–3.2	26	28	69.2	67.0	122.5	126.0	83.0	83.5
2	73	M	Isch	InMotion2	Real	5.1–9.0	31	39	63.4	60.6	117.0	120.5	74.0	75.5
3	54	F	Isch	InMotion3	Real	2.2-3.5	15	25	77.4	70.0	116.0	117.8	76.3	75.5
4	67	M	Isch	InMotion3	Real	1.2–2.8	20	26	74.8	74.6	125.0	124.9	80.6	82.6
5	26	F	Haem	InMotion3	Real	1.6–2.0	13	16	78.2	76.6	104.7	106.0	71.1	71.5
6	52	F	Haem	InMotion3	Real	2.0–7.0	14	17	85.1	84.6	105.0	106.1	71.3	73.1
7	60	M	Isch	InMotion3	Real	1.1–4.0	37	43	67.1	65.8	108.7	103.5	73.8	74.0
*Mean*	53.7					**2.0–4.5**	**22.3**	**27.7**	**73.6**	**71.3**	**114.1**	**115.0**	**75.7**	**76.5**
8	70	M	Isch	InMotion2	Sham	1.5–8.0	18	19	75.9	72.0	129.0	121.0	76.8	74.8
9	42	F	Haem	InMotion2	Sham	1.6–9.0	25	25	92.3	85.3	141.5	130.5	89.5	87.0
10	75	M	Isch	InMotion3	Sham	3.0–5.0	56	61	77.1	71.5	116.5	116.5	70.0	71.0
11	41	F	Isch	InMotion3	Sham	4.0–9.0	30	37	80.5	76.2	134.7	134.0	88.9	87.9
12	50	M	Haem	InMotion3	Sham	4.0–5.0	34	35	75.8	73.0	134.1	124.4	92.5	88.9
*Mean*	55.6					**2.8–7.2**	**32.6**	**35.4**	**80.3**	**75.6**	**131.2**	**125.3**	**83.5**	**81.9**

Isch: ischemic; Haem: haemorrhagic; FMA: Fugl–Meyer assessment; HR: heart rate; sBP: systolic blood pressure; dBP: diastolic blood pressure. PRE refers to values recorded immediately before VNS session and POST to values recorded immediately after the end of VNS session. VNS range intensity is expressed in mA.
